# Einflüsse der Coronapandemie auf gesundheitsbezogene Verhaltensweisen und Belastungen von Studierenden: Eine Erhebung an der Ostfalia Hochschule für angewandte Wissenschaften

**DOI:** 10.1007/s11553-021-00893-2

**Published:** 2021-08-23

**Authors:** Saskia Ehrentreich, Linda Metzner, Sandra Deraneck, Zlata Blavutskaya, Sandra Tschupke, Martina Hasseler

**Affiliations:** Standort Wolfsburg, Fakultät Gesundheitswesen, Ostfalia Hochschule für angewandte Wissenschaften – Hochschule Braunschweig/Wolfenbüttel, Rothenfelder Str. 10, 38440 Wolfsburg, Deutschland

**Keywords:** COVID-19, Studierendengesundheit, Studentisches Gesundheitsmanagement, Fragebogenerhebung, Bildungseinrichtung, COVID-19, Student health, Student health management, Questionnaire survey, Educational institution

## Abstract

**Hintergrund:**

Studierende bilden die größte Gruppe an Hochschulen, somit ist ein studentisches Gesundheitsmanagement unerlässlich. Diese Gruppe zählt zwar aufgrund ihres Alters zu einer eher gesunden Bevölkerungsgruppe, dennoch sind auch Studierende gesundheitlichen Belastungen ausgesetzt. Daher sind die Auswirkungen der Coronapandemie auf die Studierenden von allgemeinem Interesse.

**Fragestellung:**

Der Artikel befasst sich mit der Frage, wie die Rahmenbedingungen der Coronapandemie gesundheitsbezogene Verhaltensweisen und Belastungen der Studierenden an der Ostfalia Hochschule für angewandte Wissenschaften beeinflussen.

**Material und Methoden:**

Auf Grundlage einer Literaturrecherche wurde ein Fragebogen zu den Themenbereichen Bewegung, Ernährung, wahrgenommenes Stresserleben und Suchtmittelkonsum erstellt. Die Stichprobe der Onlinebefragung umfasst 1281 Studierende. Die Ergebnisse wurden über Microsoft Excel ausgewertet.

**Ergebnisse:**

Infolge der Coronapandemie bewegen sich Studierende in ihrem Alltag weniger, müssen aufgrund der gesetzlichen Regelungen ihre Mahlzeitenplanung neu organisieren und sind, z. B. durch veränderte Prüfungsleistungen oder den Verlust des Nebenjobs, einem höheren Stresslevel ausgesetzt. Der Suchtmittelkonsum bleibt dabei nahezu unverändert.

**Schlussfolgerung:**

Die Coronapandemie bewirkt sowohl positive als auch negative Veränderungen der gesundheitlichen Verhaltensweisen und Belastungen bei Studierenden. Dennoch sind weitere Untersuchungen erforderlich, um die Auswirkungen der Pandemie auf die Studierenden umfassender in den Blick zu nehmen.

## Abstract (en)

**Background:**

Students form the largest group at universities. Therefore, it is essential to establish a student health management concept for their health promotion. Due to their young age, students generally belong to a rather healthy population group. However, they may also suffer from health problems. Especially the effects of the Corona pandemic on the students should be considered.

**Objectives:**

The following article aimed how the context of the Corona pandemic influences health-related behaviors and stresses among students at Ostfalia University of Applied Sciences.

**Materials and methods:**

Based on a literature research, a questionnaire was created in order to assess students’ behaviors on the topics *physical activity, nutrition, perceived stress, *and *addictive substance consumption*. In all, 1281 students participated in the online survey. The results were analyzed using Microsoft Excel.

**Results:**

As a result of the Corona pandemic, students move less in their daily lives, have to reorganize their meal planning due to legal regulations, and are exposed to higher stress levels, e.g., due to changes in exam performance or the loss of a part-time job. The consumption of addictive substances remains virtually unchanged.

**Conclusions:**

The Corona pandemic is causing positive and negative changes in health behaviors and burdens among students. Nevertheless, further research is needed to look more comprehensively at the impact of the pandemic on students.

Das Coronavirus (SARS-CoV‑2 [„severe acute respiratory syndrome coronavirus 2“]) steht seit Beginn des Jahres 2020 aufgrund der schnellen Ausbreitung und Übertragung des Virus international im Fokus. Das hohe Ansteckungspotenzial sowie die infektiöse Lungenerkrankung COVID-19 („coronavirus disease 2019“) machen das Virus zu einem gesellschaftsrelevanten Thema und zeigen Auswirkungen in allen Bereichen des persönlichen und öffentlichen Lebens. Auch auf die Gesundheit von Studierenden zeigt die Coronapandemie nicht zu vernachlässigende Auswirkungen.

## Einleitung

Studierende bilden die größte Gruppe an Hochschulen, weswegen es unerlässlich ist, ein studentisches Gesundheitsmanagement als Kernelement der Gesundheitsförderung in diesen Einrichtungen zu fördern [22]. Aufgrund ihres Alters gehören Studierende insgesamt einer gesunden Bevölkerungsgruppe an [11]. Jedoch kann der Wechsel von der Schule an die Hochschule oder Universität oft essenzielle biographische Veränderungen bewirken, welche wiederum zu Verunsicherungen und folglich zu einem schlechteren Gesundheitszustand oder einem risikoreicheren Gesundheitsverhalten, z. B. in Form eines höheren Alkoholkonsums oder einer ungesünderen Ernährung, führen können [16]. Ebenso sorgt die Coronapandemie für eine Veränderung der gesundheitsbezogenen Verhaltensweisen und Belastungen der Studierenden, denn auch Hochschulen sind von den Maßnahmen zur Einschränkung des Coronavirus betroffen. So wurden das Sommersemester 2020 wie auch das Wintersemester 2020/2021 überwiegend digital durchgeführt [12]. Zudem wurden Maßnahmen erlassen, welche sich auf die Ernährung der Studierenden auswirken, da Restaurants und Mensen geschlossen wurden oder ihren Betrieb nur unter Auflagen fortführen konnten [7]. Daher mussten sich die Studierenden in ihrer Mahlzeitenplanung umstellen. Infolge der Coronapandemie begegnet eine Vielzahl der Studierenden veränderten Alltagsstrukturen [24] sowie einer höheren Arbeits- und Stundenbelastung im digitalen Studium [5, 24]. Weiterhin berichten 50 % der Studierenden der Zürcher Hochschule für angewandte Wissenschaften (ZHAW) über negative Auswirkungen auf ihre finanzielle Situation [24]. Zudem fehlen den Studierenden die sozialen Kontakte, was mit einem höheren Einsamkeitsempfinden und Spannungserleben einhergeht [24]. Im Kontext des Suchtmittelkonsums prognostizieren Experten und Expertinnen einen steigenden Konsum von Suchtmitteln [9]. Kurzfristig ist insbesondere bei jungen, bereits regelmäßig Konsumierenden mit einem Anstieg des Alkoholkonsums zu rechnen, da durch Homeoffice und eintretende Arbeitslosigkeit das strukturierte Leben abnimmt und Möglichkeiten des sozialen Kontaktes weniger ausgeschöpft werden können. Mittel- und langfristig ist jedoch eine Abnahme des Pro-Kopf-Konsums zu erwarten [18]. Im Rahmen des Tabakkonsums ist eine Steigerung des Konsums regelmäßig Rauchender, aufgrund geringerer Alltagsstrukturen und verringerter sozialer Einflussnahme, anzunehmen. Der Lockdown könnte zudem stressbedingt das Rückfallrisiko verstärken oder einen Verzögerungs- oder Vorbeugungseffekt bei Nichtrauchenden auslösen [19].

Bisher existieren jedoch insgesamt wenig abgeschlossene Untersuchungen zwischen dem Zusammenhang der Studierendengesundheit und der Coronapandemie, v. a. die Auswirkungen auf das Bewegungsverhalten der Studierenden wurden noch nicht umfassend untersucht. Aus diesem Grund beschäftigt sich der Artikel mit der Frage, wie die Rahmenbedingungen der Coronapandemie gesundheitsbezogene Verhaltensweisen und Belastungen der Studierenden an der Ostfalia Hochschule für angewandte Wissenschaften^1^ beeinflussen.

## Methodische Vorgehensweise

### Erhebungsinstrument

Auf Grundlage einer wissenschaftlichen Literaturrecherche wurde ein (voll)standardisierter Fragebogen zu den Themenbereichen *Bewegung, Ernährung, wahrgenommenes Stresserleben, Suchtmittelkonsum *und *Studium mit Kind *erstellt. Die Ergebnisdarstellung zu *Studium mit Kind* wird aufgrund einer zu geringen Stichprobe nicht weiterverfolgt. Die Auswirkungen der Coronapandemie auf die Studierendengesundheit wurden als Teilbereich einer Befragung zu den gesundheitsrelevanten Belastungen und Verhaltensweisen der Studierenden an der Ostfalia erhoben. Innerhalb der Befragung wurden Unterschiede zwischen den Zeiträumen vor und während der Pandemie erfragt. Die quantitativen Antwortmöglichkeiten wurden mit Hilfe einer Ordinalskala gestaltet. Die qualitativen Fragen konnten durch Freitextfelder beantwortet werden.

### Stichprobe

Die Stichprobe umfasst die 14.570 Studierenden der Ostfalia der Standorte Salzgitter, Suderburg, Wolfenbüttel und Wolfsburg. Es wurden keine weiteren Einschlusskriterien definiert, denn die Ergebnisse sollten unabhängig von bestimmten Kriterien erhoben und ausgewertet werden. Die quantitative Studie umfasst 1281 vollständig ausgefüllte Fragebögen.

### Durchführung der Erhebung

Die Befragung wurde online über *LimeSurvey* (Hamburg, Deutschland) durchgeführt. Die Studierenden konnten über einen Link, den sie via Hochschul-E-Mail erhielten, auf die Befragung zugreifen. Vor dem Befragungsbeginn wurden den Studierenden datenschutzrechtliche Hinweise sowie Informationen zum Befragungsablauf eingeblendet. Zudem wurde auf die Wahrung der Anonymität im Befragungsprozess verwiesen. Die Befragung wurde vom 05.11.2020 bis 03.12.2020 durchgeführt. Der bereinigte Rücklauf beträgt 8,79 % (1281 Studierende).

### Auswertung

Die Auswertung erfolgte mit Hilfe von *Microsoft Excel* (Microsoft Corporation, Redmond, WA, USA). Zu Beginn wurden die deskriptiven quantitativen Daten analysiert und prozentual ausgewertet; im Falle von Mehrfachnennungen wurden lediglich absolute Werte genutzt. Die Auswertung der qualitativen Daten erfolgte durch die schrittweise Zusammenfassung der inhaltlichen Merkmale der Antworten in Kategorien, welche analog den quantitativen Daten ausgewertet wurden. Sowohl die quantitativen als auch die qualitativen Daten wurden graphisch veranschaulicht.

## Ergebnisse

### Soziodemographische Daten

Von den 1281 Teilnehmenden sind 57,6 % weiblich, 41,8 % männlich und 0,6 % divers. 55,8 % der Befragten sind zwischen 21 und 25 Jahren alt. Hinsichtlich des Familienstands sind 92,4 % der Studierenden ledig. Die Angaben der Studierenden bezüglich des angestrebten Studienabschlusses gestalten sich folgendermaßen: Bachelor (*n* = 1104), Master (*n* = 140), keine Angabe (*n* = 37). Zudem zeigen die Ergebnisse, dass mehr als die Hälfte der Studierenden (*n* = 663) finanziell von ihren Eltern unterstützt werden. Fast ebenso viele Studierende (*n* = 619) sind während des Studiums erwerbstätig, 363 Studierende (auch) in den Semesterferien.

### Bewegung

An den Ergebnissen der Befragung ist zu erkennen, dass verschiedene Gründe dazu führen, dass Studierende sich im Alltag nicht ausreichend bewegen. Am häufigsten werden diesbezüglich die Faktoren Zeit, Motivation sowie der aktuelle Lockdown genannt. Zudem wird die Coronapandemie als häufigster Grund, sich nicht bei Sportkursen der Ostfalia angemeldet zu haben, angeführt. Die Pandemie wirkt sich negativ auf das Bewegungsverhalten von 64,7 % der Studierenden aus. Durch die Einführung von „distance learning“ fehlt 64,0 % der Teilnehmenden (eher) die Alltagsbewegung. Nur 17,6 % der Studierenden können trotz der aktuellen Situation ihren gewohnten Bewegungsmöglichkeiten nachgehen. 22,4 % der Befragten nutzen andere Bewegungsmöglichkeiten als vorher.

### Ernährung

Vor der Coronapandemie aßen 74,3 % der Studierenden im Alltag *(eher) regelmäßig*. Die Mehrheit der Befragten gibt bei den Aussagen, ob sie *mehr, regelmäßiger *oder *mehr naturbelassene Lebensmittel* essen als vor der Coronapandemie, an, dass diese auf sie *eher nicht *oder *gar nicht zutreffen*. Jedoch essen seit der Coronapandemie 7,1 % der Studierenden *mehr *und 25,4 %* eher mehr als vorher*. Außerdem essen seit der Coronapandemie 6,9 % der Teilnehmenden *regelmäßiger *und 21,9 % der Studierenden *eher regelmäßiger als vorher*. Weiterhin geben 5,2 % der Befragten an, seit der Pandemie *mehr naturbelassene Lebensmittel zu essen als vorher *und auf 19,9 % der Studierenden *trifft* diese Aussage *eher zu*. Bei der Inanspruchnahme von Lieferservices seit der Coronapandemie im Vergleich zu vor dem Ausbruch zeigen die Ergebnisse einen Anstieg der Häufigkeit der Nutzung. Dies ist an häufigen Angaben wie *mehrmals pro Woche, einmal pro Woche* und *mehrmals im Monat* zu erkennen. Entgegengesetzt dazu zeigen die Ergebnisse zur Inanspruchnahme von Restaurants und Imbissen seit der Coronapandemie im Vergleich zu vor dem Ausbruch einen Rückgang der Nutzung.

### Wahrgenommenes Stresserleben

Zum Befragungszeitpunkt ergab sich bei den Teilnehmenden mit Blick auf das Wintersemester 2020/2021 ein hohes Stresslevel, wonach sich 76,0 % der Befragten *(eher) gestresst *fühlten. 39,6 % der Studierenden nehmen die coronabedingte Studiensituation seit dem Sommersemester 2020 *stressiger *wahr als das Studium vor Pandemiebeginn. In der Prüfungsphase im Sommersemester 2020 fühlten sich 54,6 % der Befragten *(eher) gestresst*. Die letzte coronafreie Prüfungsphase im Wintersemester 2019/2020 empfanden 34,0 % der Studierenden – im Vergleich zur Coronaprüfungsphase im Sommersemester 2020 – als *gleichbleibend stressig*. 26,5 % der Befragten hingegen nahmen die coronafreie Prüfungsphase als *weniger stressig *wahr und 13,3 % wiederum als *stressiger*.

Aufgrund der Auswirkungen der Coronapandemie erfuhren 18,9 % der Befragten (242 Studierende) einen Verlust oder eine Stundenreduktion ihrer Nebenbeschäftigung. Von jenen 242 Studierenden berichten 66,1 % sich durch diesen Umstand *(eher) gestresst* zu fühlen. Darüber hinaus löst insbesondere die Einschränkung der sozialen Kontakte ein mehrheitlich hohes Stressempfinden der Studierenden aus: 69,4 % fühlen sich infolgedessen *(eher) gestresst*. Ebenso der coronabedingt höhere Anteil an Selbstorganisation verstärkt das Stresserleben der Studierenden – 56,7 % fühlen sich *(eher) gestresst*. Auch im Zuge folgender Veränderungen ergibt sich eine nicht zu vernachlässigende, aber nicht überwiegende Stresswahrnehmung der Studierenden: die Neuorganisation des Alltags, der Übergang zum digitalen Studium sowie die Verschiebung von Studien- und Abschlussarbeiten. Eine von den Befragten getroffene Gesamteinschätzung des Umgangs mit der Coronapandemie zeigt, dass 10,1 % die Veränderungen *gut* und 46,6 % *eher gut* bewältigen; 32,0 % bewerkstelligen die Situation hingegen *eher schlecht *und 10,1 %* schlecht*.

Auch in den von den Studierenden wahrgenommenen, größten Stressfaktoren im Studium spiegeln sich Auswirkungen der Coronapandemie wider. So bestätigt sich das hohe Stresserleben aufgrund der eingeschränkten sozialen Interaktion (105 Studierende). Weiterhin werden u. a. Online-Veranstaltungen (56 Studierende), Motivationsschwierigkeiten im digitalen Studium (48 Studierende) sowie die fehlende neutrale Studienumgebung (40 Studierende) als stressfördernd bewertet.

### Suchtmittelkonsum

Im Rahmen des Suchtmittelverhaltens der Studierenden zeigen sich folgende Tendenzen im Zuge der Coronapandemie: der Suchtmittelkonsum bleibt überwiegend unverändert oder nimmt tendenziell ab. Zum Thema Rauchverhalten berichten 68,3 % über einen unveränderten Konsum, 20,1 % über einen weniger ausgeprägten Konsum. Darüber hinaus geben 42,5 % der Studierenden einen unveränderten und 27,0 % einen geringeren Alkoholkonsum an. Ebenso bleibt der illegale Substanzkonsum überwiegend unverändert (79,1 %) und ging bei 15,5 % der Studierenden zurück. Zuletzt zeigt sich diese Entwicklung auch im Rahmen des Neuroenhancements: 85,9 % der 1281 Befragten geben ein unverändertes Neuroenhancement an, 12,3 % berichten über einen geringeren Konsum leistungssteigernder Mittel.

## Diskussion

Im Rahmen dieser Erhebung wurden die Auswirkungen der geänderten Rahmenbedingungen aufgrund der Coronapandemie auf die gesundheitsbezogenen Verhaltensweisen und Belastungen der Studierenden an der Ostfalia untersucht. Seit dem Ausbruch der Coronapandemie bewegen sich knapp zwei Drittel der Befragten weniger. Obwohl keine expliziten Gründe für die Bewegungsreduktion erhoben wurden, ist zu vermuten, dass hierzu Einschränkungen infolge der Pandemie beitragen. Wie die Untersuchung zeigt, fehlt seit der Einführung von „distance learning“ der Mehrheit der Studierenden der Ostfalia die alltägliche Bewegung. Lediglich 17,6 % der Studierenden können ihren gewohnten Bewegungsmöglichkeiten nachgehen und 22,4 % nutzen andere Bewegungsmöglichkeiten. Aufgrund der Beschlüsse der Regierung sind viele Möglichkeiten der Sportausübung sowie die Bewegungsfreiheit eingeschränkt. So beeinflusst die Schließung der öffentlichen und privaten Sportbetriebe und das Verbot der Zusammenkünfte in Vereinen und sonstigen Sport- und Freizeiteinrichtungen das Bewegungsverhalten. Somit bleibt den Studierenden die Möglichkeit, sich im Freien oder in der eigenen Wohnung zu bewegen. Darüber hinaus trägt der Aufruf der Regierung, nach Möglichkeit zu Hause zu bleiben [3], dazu bei, dass alltagsübliche Bewegungsmöglichkeiten wie Spaziergänge unterlassen werden. Eine Studie von Chaturvedi et al. berichtete, dass die Lockdown-bedingte Veränderung der Fitnessroutine einen Einfluss auf das allgemeine gesundheitliche Wohlbefinden hatte [4].

Auch im Ernährungsverhalten der Studierenden spiegeln sich Auswirkungen des Lockdowns wider. Die Nutzung von Restaurants und Imbissen ist seit dem Ausbruch der Coronapandemie stark zurückgegangen, was z. T. dadurch bedingt ist, dass zur Eindämmung der Pandemie Restaurants schließen mussten. Viele Restaurants und Imbisse sind auf das eingeschränkte Angebot des Essens zum Mitnehmen umgestiegen, da das Essen nicht vor Ort verzehrt werden darf. Hiermit sind alltagsübliche Aktivitäten (wie Essen außer Haus mit Freunden, Familie oder Geschäftsessen) unmöglich. Des Weiteren ist ein Anstieg der Inanspruchnahme der Lieferdienste, durch den längeren Aufenthalt zu Hause, zu verzeichnen. Aufgrund des „distance learning“ und der für viele erwerbstätige Studierende geltenden Anordnung zum Homeoffice ist anzunehmen, dass einigen Studierenden nicht ausreichend Zeit zum Kochen bleibt, weswegen auf Lieferdienste zurückgegriffen wird. Die Studierenden geben u. a. an, dass sie mehr essen, da das Essen zu Hause immer verfügbar ist. Andererseits verzehren einige Studierende mehr naturbelassene Lebensmittel und ernähren sich regelmäßiger als vor der Pandemie. Auch hier spiegelt sich vermutlich der Faktor, dass mehr Zeit zu Hause verbracht wird und die damit verbundene zeitliche Flexibilität wider. Ein weiterer Punkt ist die eingeschränkte Möglichkeit der Außer-Haus-Verpflegung sowie die Einschränkung der sozialen Kontakte, wodurch Studierende sich häufiger selbst verpflegen müssen, indem sie ihre Mahlzeiten eigenständig planen, organisieren und gegebenenfalls zubereiten müssen.

In der Coronaprüfungsphase im Sommersemester 2020 fühlten sich 54,6 % der Befragten (eher) gestresst. Weiterführend bewerten 26,5 % der Befragten die coronafreie Prüfungsphase als weniger stressig im Vergleich zur Coronaprüfungsphase. Diesbezüglich sind folgende Gründe naheliegend: Viele Studierende mussten neuartige Prüfungssituationen wie Online- oder Kombinationsprüfungen in der Coronaprüfungsphase ablegen. Ebenfalls berichten die Studierenden über erschwerte Kommunikations- und Austauschmöglichkeiten. Als Ursache für die Umstellung auf die seit dem Sommersemester 2020 eingeführte Online-Lehre gelten die Einschränkungen der Coronapandemie. Die Online-Lehre selbst stellt einen weiteren relevanten Stressfaktor dar, welcher auch in einer Studie von Sundarasen et al. als einer der entscheidendsten Stressfaktoren erhoben wurde [20]. Obwohl mehr als die Hälfte der Studierenden mit der Coronasituation insgesamt gut bis sehr gut zurechtkommen, liegt der Anteil der Studierenden, die schlecht mit der Situation umgehen können, bei 10,1 %. Ähnliche Ergebnisse liefert auch die ZHAW [24]. Mit Hilfe der in der Befragung erhobenen Daten wird deutlich, dass die Einschränkungen der sozialen Kontakte ebenfalls für das Stresserleben der Studierenden relevant sind. So sind 69,4 % der Studierenden durch das Fehlen der sozialen Kontakte (eher) gestresst. Der Aspekt der fehlenden sozialen Kontakte bestätigt sich ebenfalls in Befragungen anderer Universitäten [13, 17, 24]. Eine Befragung an der Universität Siegen ergab, dass die Coronapandemie und der damit einhergehende Lockdown bei Studierenden Stress und Ängste auslöst, aufgrund des Verfolgens der Infektionszahlen, des Lebens im Lockdown und der reichlichen Zeit allein vor dem Computer. Sorgen und Ängste der Studierenden wurden ebenfalls durch ein erhöhtes Arbeitspensum, technische Probleme hinsichtlich der Online-Lehre und der Selbstdisziplin ausgelöst [13]. Außerdem berichten Studierende über die Schwierigkeit, eine Balance zwischen Studium und Freizeit zu finden. Aufgrund einer fehlenden räumlichen Trennung zwischen Alltag und Studium, fällt es einigen Studierenden schwer, gedanklich abzuschalten. Dies wird verstärkt, wenn Studierende zusätzlich ihren (Neben)job im Homeoffice ausüben. Nach Son et al. geht die Pandemie darüber hinaus mit Konzentrationsschwierigkeiten und einer Abnahme der akademischen Leistungen einher [17]. Die in der Befragung erhobenen Motivationsschwierigkeiten, die viele Studierende der Ostfalia während des Online-Studiums wahrnahmen, bestätigten sich auch in einer Studie von Browning et al.: Demnach verspürten 21,5 % der 14.174 Befragten Motivationslosigkeit während der Coronapandemie [2]. Auch die Sorge der Studierenden, insbesondere für Risikopatient*innen, sich mit dem SARS-CoV-2-Virus zu infizieren, ist ein weiterer relevanter Stressfaktor [6]. Dies bestätigt sich ebenfalls in einer Studie von Son et al., wonach jedoch nicht nur die eigene Gesundheit bei den Studierenden Stress auslöst, sondern auch die der Angehörigen [17]. Auch wenn der Stress bei 71,3 % der 2031 Studierenden während der Pandemie ansteigt, berichten 43,3 % diesen Stress bewältigen zu können [23]. Eine Studie von Fu et al. konnte allerdings keine signifikanten Unterschiede im Stresserleben zwischen den weiblichen und männlichen Studierenden feststellen [10]. Zu ähnlichen Ergebnissen kam eine Studie von Omar und Oksana [14]. Abuhmaidan und Al-Majali hingegen ermitteln, dass weibliche Studierende während der Coronapandemie ein geringeres Level psychischer Gesundheit vorwiesen als männliche Studierende [1]. Weibliche Studierende weisen pandemiebedingt ein höheres Level an Depressionen, Ängsten, Einsamkeit und Stress auf, wobei keine signifikanten Unterschiede zwischen den Geschlechtern festgestellt werden konnten [8]. Jedoch zeigen Super und Van Disseldorp, dass zwischen den Geschlechtern Differenzen in der mentalen Gesundheit bestehen [21]. Vergleichend zeigt eine weitere Studie, bezogen auf die deutsche Gesamtbevölkerung, dass 30 % der Frauen die Coronazeit als weniger stressig empfinden, bei Männern liegt der Wert bei 24 % [15]. Hingegen sind knapp 20 % der Frauen und 15 % der Männer der Meinung, dass ihr Stresslevel seit Ausbruch der Pandemie gestiegen ist [15].

Die stressbezogene Belastung der Studienanfänger*innen fiel niedriger aus als bei den Studierenden der höheren Semester. Als Grund dafür wird das geänderte Curriculum genannt, wodurch die Studienanfänger*innen kein Praktikum absolvieren müssen [10].

Obwohl Experten und Expertinnen von einer Zunahme des Suchmittelkonsums als Folge der Coronakrise ausgehen [9], konnte dies durch die Befragung nicht bestätigt werden. Bei den Studierenden der Ostfalia bleibt das Verhalten in allen Kategorien überwiegend unverändert, in einigen Kategorien ist eine Abnahme des Konsums zu verzeichnen. Es lässt sich vermuten, dass die Einschränkungen des sozialen Lebens eine Ursache hierfür bilden. Durch die Gesetzesauflagen zur Eindämmung des Coronavirus sind Treffen in größeren Gruppen, Restaurantbesuche sowie Feierlichkeiten unmöglich, was eine Erklärung für den Rückgang des Suchtmittelkonsums darstellen kann.

## Limitationen

Aufgrund der Erhebung von unterschiedlichen Zeiträumen vor und während der Pandemie kann ein „recall bias“ nicht ausgeschlossen werden. Dies kann Verzerrungen in der Erhebung zur Folge haben. In der Befragung zur Studierendengesundheit an der Ostfalia werden personenbezogene, z. T. sensible, Daten abgefragt. Unter der Berücksichtigung des Phänomens sozial erwünschten Antwortens besteht keine Sicherheit, dass alle Angaben der Wahrheit entsprechen. Da die Teilnahme an der Studierendenbefragung freiwillig war, könnte in der Stichprobe der Selektionsbias auftreten. Die Motivation zur Teilnahme konnte durch eine sehr positive oder sehr kritische Grundhaltung zum gesamten Thema des Gesundheitsmanagements der Studierenden beziehungsweise zu einzelnen Themenbereichen beeinflusst sein.

## Ausblick

Die Befragungsergebnisse veranschaulichen vielfältige Auswirkungen der Coronapandemie auf die Lebenswelt der Studierenden. So sind sowohl positive als auch negative Einflüsse innerhalb der befragten Themenbereiche ersichtlich. Dennoch sind weiterführende Erhebungen zu den Auswirkungen der Coronapandemie unerlässlich, um ein umfassenderes Bild über die Pandemieauswirkungen auf die Studierenden zu erhalten. Im Rahmen des studentischen Gesundheitsmanagements ist es in diesen Zeiten erforderlich, sich in der Maßnahmen- und Angebotsbildung an die Rahmenbedingungen der Coronapandemie anzupassen. So sind Angebote zum Gesundheitsverhalten sowie zur Belastungsbewältigung auf digitale Formate umzustellen, um Studierende auch in Krisenzeiten entsprechend zu unterstützen. Zudem sollten Hilfsangebote für Studierende in finanziellen Notlagen etabliert werden. Da unklar ist, wann ein normaler Hochschulbetrieb wieder möglich sein oder ob in Zukunft auf Hybridmodelle gesetzt wird, sind Maßnahmen auf struktureller Ebene der Hochschulen unerlässlich.

## Fazit für die Praxis

- Die Coronapandemie schafft neuartige gesundheitliche Belastungen und kann negative gesundheitsbezogene Verhaltensweisen verstärken.
- Insbesondere die Umstellung auf die Online-Lehre sowie damit einhergehende Veränderungen, z. B. im Rahmen von Prüfungsleistungen, erhöhen das Stresslevel von Studierenden.
- Langfristig kann eine gezielte Anpassung des Hochschulwesens an die coronabedingten Rahmenbedingungen die gesundheitliche Situation der Studierenden verbessern.

## Footnotes

1 Wird fortlaufend als Ostfalia bezeichnet.
